# Exploring minocycline’s effect on retinal degeneration following *N*-methyl-*N*-nitrosourea exposure in rats

**DOI:** 10.17221/122/2024-VETMED

**Published:** 2025-07-25

**Authors:** Burak Karabulut, Hatice Eroksuz, Yesari Eroksuz, Mehmet Gul

**Affiliations:** ^1^Department of Pathology, Veterinary Faculty, Firat University, Elazig, Turkiye; ^2^Department of Histology, Medicine School, Inonu University, Malatya, Turkiye

**Keywords:** minocycline, retinal degeneration, retinitis pigmentosa

## Abstract

Retinal degeneration (RD) is often associated with deficiencies or the inaccurate production of photoreceptor-specific proteins, which are encoded by various genes and characterised by the apoptotic and ongoing death of photoreceptor cells. This study involved administering a single intraperitoneal (i.p.) dose of 50 mg/kg of *N*-methyl-*N*-nitrosourea (MNU) to rats to induce RD. Some of these rats also received intraperitoneal minocycline at varying doses to prevent RD. Euthanasia was conducted at five intervals: at 12, 24, 48, and 72 h, and on the 7^th^ day; and eye samples were taken. These samples were analysed using histopathology, immunohistochemistry, and electron microscopy. Significant RD was observed in the MNU-treated groups, with photoreceptor cell apoptosis demonstrated by the TUNEL method. Compared with those in the control group, there was a progressive thinning of the photoreceptor layer and outer nuclear layer, along with increased levels of glial fibrillary acidic protein (GFAP) and proliferating cell nuclear antigen (PCNA), and reduced levels of rhodopsin and red/green opsin starting from the 12^th^ hour in the experimental groups. Electron microscopy revealed that amacrine and bipolar cells, in addition to photoreceptors, were also affected. The minocycline treatment did not show significant differences in retinal layer thickness or the staining levels of PCNA, GFAP, and opsins in the MNU-induced RD model.

Retinitis pigmentosa, diabetic retinopathy, retinal detachment, and macular degeneration are the most common retinal diseases and causes of blindness in humans (Yang et al. 2009). Similar conditions, such as sudden acquired retinal degeneration in dogs (Lynch et al. 2023), progressive retinal atrophy in cats (Minella et al. 2018), and outer retinal degeneration in monkeys (Kosec et al. 2020), have been reported. These diseases are marked by retinal degeneration (RD), characterised by the ongoing death of the photoreceptors, which leads to blindness in about 50% of cases (Aplin et al. 2014).

The development of retinal disorders involves the massive degeneration and death of photoreceptor cells. First, the degeneration of rod receptors, responsible for monochromatic vision, is followed by the degeneration of cone receptors, which are responsible for polychromatic vision, and this is the main cause of vision loss (Sun et al. 2023).

Experimental models are crucial to understanding the mechanisms and developing treatment strategies for retinal disorders. These models can be generated through genetic techniques such as transgenic animals, exposure to intense light, or the use of specific chemicals (Emoto et al. 2014).

*N*-methyl-*N*-nitrosourea (MNU) is an alkylating compound that shows cytotoxic effects by transferring methyl groups to nucleobases and is a suitable candidate for RD models due to its selective impact on photoreceptor cells (Yan et al. 2024). The exact reason for this selectivity is not fully understood, but one possible explanation is the lower glutathione levels in photoreceptor cells. Glutathione is known to metabolise MNU and remove alkylation products (Tappenier et al. 2013).

Tetracyclines are broad-spectrum antibiotics with bacteriostatic effects. Minocycline, a second-generation, long-acting, semi-synthetic antibiotic, has been shown in numerous studies to inhibit matrix metalloproteinases, tumour-induced angiogenesis, nitric oxide synthase, caspase-3, and the release of oxygen radicals from neutrophils, independent of its antimicrobial properties (He et al. 2021). These attributes make minocycline a potential inhibitor for the research of apoptosis-related retinal degeneration.

## MATERIAL AND METHODS

### Animal experiment

This experiment was conducted with 100 female Sprague Dawley rats, two months old and weighing between 150 and 200 grams, sourced from the Firat University Research Centre. Ethical approval was granted by the Firat University Local Ethics Committee (09.03.2016, Decision No. 46). The rats were separated into five groups, each consisting of 20 animals. One group was designated as the control, and the remaining four were the experimental groups. The first experimental group was the MNU group, the second was the MNU and low-dose minocycline (LDM) group, the third was the MNU and medium-dose minocycline (MDM) group, and the fourth was the MNU and high-dose minocycline (HDM) group.

The animal study was conducted in standard cages under normal conditions of humidity and temperature, with unrestricted feeding and no special conditions applied. The rats in the experimental groups (MNU, LDM, MDM, and HDM) were administered a single dose of MNU via i.p. injection (Pfaltz & Bauer Inc., Waterbury, CT, USA) at a dosage of 50 mg/kg of body weight. The therapeutic groups (LDM, MDM, HDM) received two doses of minocycline: 24 h before and 1 h after the MNU administration, at doses of 50, 75, and 100 mg/kg of body weight, respectively. Minocycline was prepared as a 2% solution in distilled water. The control animals were given only an i.p. injection of saline.

To observe the time-dependent pathological changes, euthanasia was performed at five different time points: at 12, 24, 48, and 72 h and on the 7^th^ day. In each period, four animals from each group, totalling 20 animals, were euthanised. Animals were euthanised by decapitation after being under deep anaesthesia via the i.p. administration of 30 mg/kg of a xylazine solution, Xylazinbio (Bioveta A.S., Ankara, Türkiye), and 150 mg/kg of a ketamine solution, Brema (Bremer Pharma GmbH, Warburg, Germany). The dorsal and ventral parts of the cornea were marked with a lightly heated needle to determine the eye’s vertical plane during the trimming process. After the eyeballs were removed whole, they were fixed in Davidson’s solution (McKay et al. 2009) for about 36 hours. At each euthanasia interval, one eye from each group was fixed in a 2.5% glutaraldehyde solution for electron microscopic examination.

### Tissue preparation

The eye specimens, fixed in Davidson’s solution, were cut into two halves at the midline (Reisenhofer et al. 2015), using the previously marked corneal scars as a guide, and the lens was removed with forceps. The eye halves were then placed in standard tissue processing cassettes (Isolab GmbH, Wertheim, Germany). After being washed under running tap water for about two hours, the samples were processed through a series of graded alcohols, xylene, and paraffin in an automatic tissue processing device (TP 1020; Leica, Wetzlar, Germany), and subsequently embedded in paraffin using a tissue embedding device (EG 1150 H; Leica, Wetzlar, Germany). Histological sections were taken using a rotary microtome (RM2125; Leica, Wetzlar, Germany) and placed on positively charged slides (Superfrost; Thermo Fisher Scientific, Waltham, MA, USA).

### Morphometric analysis of retinal layers

**Figure 1:**
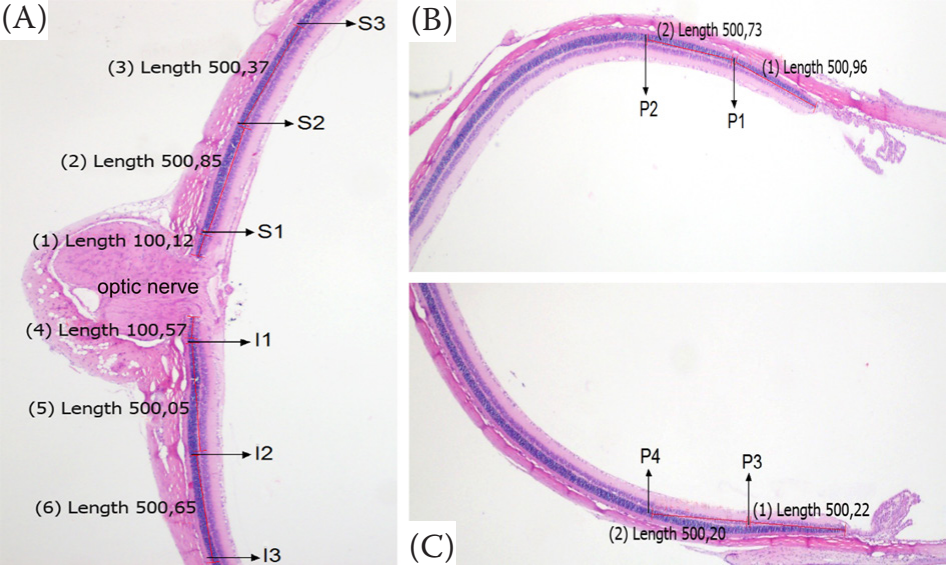
Photoreceptor layer and outer nuclear layer measurement points (μm) in the central retina (A) and peripheral retina (B–C) (B) Superior quarter (P1 and P2); (C) Inferior quarter (P3 and P4) S1, S2, S3 = superior quarter; I1, I2, I3 = inferior quarter

Sections were stained with haematoxylin-eosin using an automatic tissue staining machine (Autostainer XL; Leica, Wetzlar, Germany) and analysed with a light microscope (BX43; Olympus, Tokyo, Japan). The photoreceptor layer (PL) and the outer nuclear layer (ONL) were measured in both the central and peripheral retina using an imaging analysis system (cellSens Standard, Tokyo, Japan). Measurements in the central retina were performed at a total of six points, three each in the superior and inferior quarters, starting at 100 μm away from the optic nerve and 500 μm apart (Zhang et al. 2004). In the peripheral retina, measurements were at distances of 500 μm and 1 000 μm from the *ora serrata* at two points in both the superior and inferior quarters, totalling four points (Figure 1).

### Immunohistochemistry

Immunohistochemical analyses were conducted using the Streptavidin Biotin Peroxidase Complex technique, following a previously described method (Hsu et al. 1981). The chemicals from a ready-to-use immunohistochemistry kit were used in accordance with the manufacturer’s standard procedure (Ultra Vision Detection System; Thermo Fisher Scientific, Waltham, MA, USA). The primary antibodies were: rhodopsin (Monoclonal, mouse; Santa Cruz, Texas, USA), red-green opsin (Polyclonal, rabbit; Merck KGaA, Darmstadt, Germany), Glial Fibrillary Acidic Protein (GFAP) (Monoclonal, mouse; Santa Cruz Biotechnology, Dallas, TX, USA) and Proliferating Cell Nuclear Antigen (PCNA) (Monoclonal, mouse; Santa Cruz Biotechnology). The slides were examined under a microscope and scored according to a previously described scoring system (Carter et al. 2004) (Table 1).

**Table 1:** Immunoscore table

Stained areas		Stain intensity
0.1: <25%!		0: negative
0.4: between 26–50%		0.5: trace
0.6: between 51–75%		1: light
0.9: between 76–100%		2: moderate
–		3: intense

Immunoscore: stained area value × stain intensity value

### TUNEL method

Apoptotic cells were identified using the Terminal Deoxynucleotidyl Transferase dUTP Nick End Labelling (TUNEL) assay with an ApopTag Plus Apoptosis Detection Kit (Merck KGaA, Darmstadt, Germany), following the protocol provided by the manufacturer. The slides were examined under a normal light microscope. To assess the degree of apoptosis, brown-stained photoreceptor cell nuclei were counted at ×400 magnification in two areas located 400 μm to the right and left (superior and inferior quarters) of the optic nerve in each eye. All the stained and unstained photoreceptor cell nuclei in the same region were then counted, and the apoptotic index was calculated as a percentage (Apoptotic index = TUNEL-positive cell count/total cell count) (Yoshizawa et al. 1999).

### Electron microscopy

Eye samples were fixed with 2.5% glutaraldehyde and 1% osmium tetroxide (Merck KGaA, Darmstadt, Germany) in an automated electron microscopic tissue processing device (EM AMV; Leica, Wetzlar, Germany). The samples were then dehydrated with acetone, embedded in araldite blocks, and polymerised. The retinal tissue surface and boundaries within the araldite blocks were exposed using a trimming device (EM TRIM; Leica, Wetzlar, Germany), and 80 nm thick sections were cut and placed on copper grids, using an ultramicrotome (Ultracut R; Leica, Wetzlar, Germany). The sections were stained with uranyl acetate and lead citrate, then analysed using a transmission electron microscope (Libra 120; Zeiss, Oberkochen, Germany).

### Statistical analysis

A statistical analysis of all the data was performed using GraphPad Prism [v10.4.2. (633); GraphPad Software, Boston, MA, USA]. The Shapiro-Wilk test was used to determine if the data conformed to a normal distribution. The Bartlett test was used to analyse whether the variances in normally distributed data were homogeneous. A one-way analysis of variance (ANOVA) was used to evaluate the data with homogeneous variances and normally distributed parameters. For pairwise group comparisons, Tukey’s test was then used. The Welch ANOVA and the post hoc Dunnett T3 test were used to assess the inhomogeneous data. Data without normal distribution were analysed with the Kruskal-Wallis and Dunn test for pairwise comparisons. The threshold for statistical significance was set at *P* < 0.05. The mean ± standard deviation was used to express the results.

## RESULTS

### Histopathological findings

**Figure 2:**
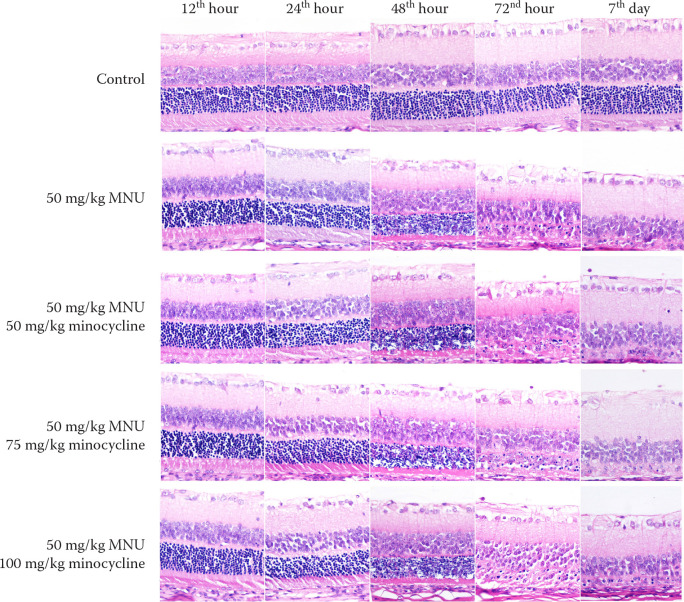
Time-dependent change of the retinal layers Increased thinning in the ONL and PL in all the groups receiving MNU MNU = *N*-methyl-*N*-nitrosourea; ONL = outer nuclear layer; PL = photoreceptor layer

The mean and standard deviation values of the ONL and PL measurements in the central retina for all the euthanasia periods are presented in Tables 2 and 3, and the time-dependent changes are illustrated in Figure 2. At the 12^th^ hour, no significant histopathological findings were observed. By the 24^th^ hour, the ONL thickness had decreased an average of 5–9 μm, and the PL thickness had been reduced by 1–2 μm in the central retinas of all the MNU-treated experimental animals. Significant differences in the ONL and PL were observed only in the central retina between the control and all the other groups treated with MNU (*P* < 0.05).

**Table 2:** The effects of the minocycline used in three different doses (50, 75 and 100 mg/kg) in the experimental retinal degeneration induced by *N*-methyl-*N*-nitrosourea (MNU) in the rats on the central outer nuclear layer thickness

Groups	Time					*P*-value
	12^th^ hour	24^th^ hour	48^th^ hour	72^nd^ hour	7^th^ day	
Control	41.82 ± 2.60	41.17 ± 3.63^A^	40.36 ± 3.93^A^	41.79 ± 1.83^A^	41.27 ± 1.41^A^	0.474**
MNU	39.88 ± 2.23^a^	34.17 ± 3.69^ab^	29.03 ± 2.76^C,b^	10.31 ± 9.27^B,c^	4.48 ± 5.17^B,c^	0.000 1***
LDM	40.48 ± 4.00^a^	32.17 ± 3.21^B,b^	31.90 ± 2.60^B,b^	14.80 ± 11.20^B,c^	4.53 ± 9.06^B,c^	0.000 1***
MDM	39.99 ± 2.90^a^	32.58 ± 3.05^B,ab^	29.78 ± 3.78^BC,b^	11.33 ± 7.39^B,c^	10.19 ± 11.02^B,c^	0.000 1***
HDM	41.33 ± 3.10^a^	32.65 ± 4.01^B,ab^	26.47 ± 2.63^D,b^	12.07 ± 8.44^B,c^	8.02 ± 7.68^B,c^	0.000 1^***^
*P*-value	0.091^***^	0.000 1^*^	0.000 1^*^	0.000 1^***^	0.000 1^***^	–

Data are given as the mean ± standard deviation

^a–c^The difference between the mean values showing different words in the same line is statistically significant (*P* < 0.05); ^A–D^The difference between the mean values showing different words in the same column is statistically significant (*P* < 0.05); *Data were evaluated with a one-way ANOVA and Tukey’s test was used as the post hoc test for the pair comparisons of the groups; **Data were evaluated with Welch’s-ANOVA and Dunnett’s T3 was used as the post hoc test for the pair comparisons of the groups; ***Data were evaluated with the Kruskal-Wallis test and Dunn’s test was used as the post hoc test for the pair comparisons of the groups

HDM = high-dose minocycline; LDM = low-dose minocycline; MDM = medium-dose minocycline

**Table 3:** The effects of minocycline used in three different doses (50, 75 and 100 mg/kg) in experimental retinal degeneration induced by *N*-methyl-*N*-nitrosourea (MNU) in rats on central photoreceptor layer thickness

Groups	Time					*P*-value
	12^th^ hour	24^th^ hour	48^th^ hour	72^nd^ hour	7^th^ day	
Control	19.13 ± 1.66^B,ab^	19.87 ± 2.36^A,a^	18.16 ± 1.96^A,b^	20.01 ± 1.99^A,a^	19.40 ± 1.32^A,ab^	0.008 3*
MNU	19.22 ± 1.37^B,a^	18.27 ± 2.23^AB,a^	11.06 ± 2.68^B,b^	2.96 ± 5.37^B,bc^	1.59 ± 5.08^B,c^	0.000 1***
LDM	19.95 ± 2.86^AB,a^	17.34 ± 1.67^B,a^	11.77 ± 2.33^B,b^	6.33 ± 6.31^B,bc^	1.04 ± 3.71^B,c^	0.000 1***
MDM	20.27 ± 2.06^AB,a^	18.92 ± 2.14^AB,a^	11.44 ± 1.94^B,b^	4.70 ± 5.98^B,b^	4.53 ± 7.37^B,b^	0.000 1***
HDM	21.54 ± 1.25^A,a^	17.90 ± 2.69^B,a^	11.32 ± 1.16^B,b^	5.79 ± 5.37^B,bc^	1.81 ± 4.30^B,c^	0.000 1***
*P*-value	0.000 1**	0.002*	0.000 1**	0.000 1***	0.000 1***	–

Data are given as the mean **±** standard deviation

^a–c^The difference between mean values showing different words in the same line is statistically significant (*P* < 0.05); ^A–D^The difference between the mean values showing different words in the same column is statistically significant (*P* < 0.05); *Data were evaluated with a one-way ANOVA and Tukey’s test was used as the post hoc test for the pair comparisons of the groups; **Data were evaluated with Welch’s-ANOVA and Dunnett’s T3 was used as the post hoc test for the pair comparisons of the groups; ***Data were evaluated with the Kruskal-Wallis test and Dunn’s test was used as the post hoc test for the pair comparisons of the groups

HDM = high-dose minocycline; LDM = low-dose minocycline; MDM = medium-dose minocycline

**Figure 3:**
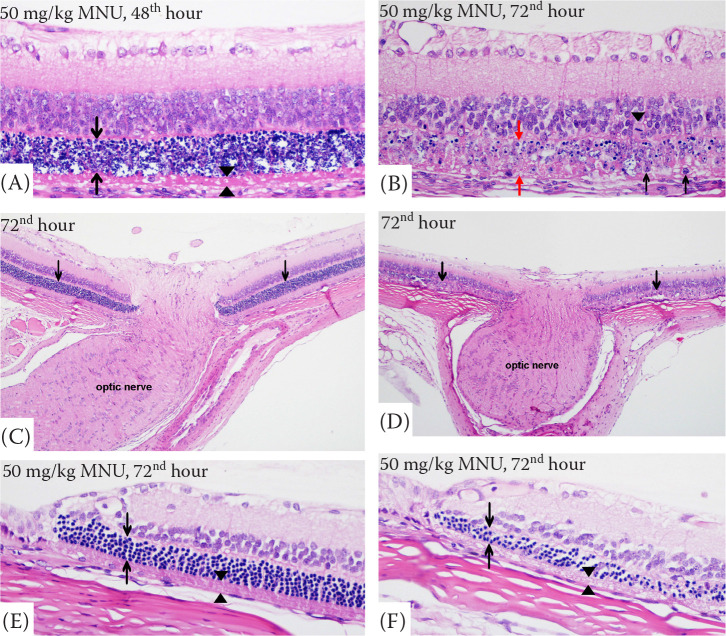
(A) Severe karyorrhexis in all the experimental groups, characterised by the disruption of the general histological structures of the ONL (region between arrows) and PL (region between the arrow heads). (B) Severe thinning and loss of borders of the ONL and PL (region between the red arrows), cellular infiltrations of macrophages (black arrows), and a mitotic figure in a Müller cell (arrowhead). (C–D) General view of the retina in the optic head at the 72^nd^ hour, normal retina (C) and thinned ONL (D) (arrows). (E–F) Normal peripheral retina (E) and thinned peripheral retina (D), ONL (regions between arrows) and PL (regions between the arrow heads) MNU = *N*-methyl-*N*-nitrosourea; ONL = outer nuclear layer; PL = photoreceptor layer

At the 48^th^ hour, the most notable histopathological change was severe karyorrhexis in all the experimental groups, marked by a disruption in the general histological structures of the ONL and PL. The fragmentation of the photoreceptor cell nuclei in the ONL was particularly characteristic at the 48^th^ hour (Figure 3A). The photoreceptor cell bodies in the PL lost their elongated, linear structure and light pink colour and showed a hypereosinophilic appearance. In the internal nuclear layer (INL), the nucleoli of retinal glia cells stained more intensely in the experimental group than in the control group. These findings were consistent across all the MNU-treated experimental groups. The mean ONL thickness in the central retinas of all the experimental groups decreased by 10–14 μm, and the PL thickness by 6–7 μm, relative to the control group. In the peripheral retina, the decrease was limited to 2–3 μm in both layers. Differences were seen in the mean ONL and PL thicknesses of the central retina between the control and experimental groups (*P* < 0.05).

At the 72^nd^ hour, the most significant histopathological finding was the severe thinning of the ONL and PL. All experimental groups had cellular infiltrations of the Müller cells and macrophages in the ONL and PL (Figure 3B), with the most intense period of these infiltrations recorded at 72 hours. Mitotic cells were present in the INL, ONL, and PL in all the experimental groups. The mean ONL thickness decreased by 25–30 μm, and the PL thickness decreased by 14–16 μm compared to the control group. In the peripheral retina, the decrease was limited to 5–9 μm in both layers. It was observed that the areas where the ONL and PL were thinnest, were closest to the optic nerve, and that the thickness of the ONL and PL returned to normal when moving approximately 1 500–2 000 micrometres away from the optic nerve. However, in three animals included in the MNU, MDM, and HDM groups, the severe thinning of the peripheral retina was observed (Figure 3E,F). A significant difference was noted between the control and experimental groups in the mean ONL and PL thicknesses in the central retina (*P* < 0.05). There was no significant difference between the experimental groups.

By the seventh day, in the experimental groups, the ONL was severely thinned due to the ongoing death of the photoreceptor cells. Complete destruction was observed in some parts of the retinas. Similarly, advanced thinning and complete disappearance were noted in some areas of the PL. The mean ONL thickness decreased by about 30–35 μm, and PL thickness by 15–17 μm compared to the control group. In the peripheral retina, the decrease was limited to 5–9 μm in both layers. There was a difference between the control and experimental groups in the central and peripheral retina thicknesses of both ONL and PL, with notable thinning in the experimental groups (*P* < 0.05). No significant differences were found between the experimental groups.

### TUNEL findings

**Figure 4:**
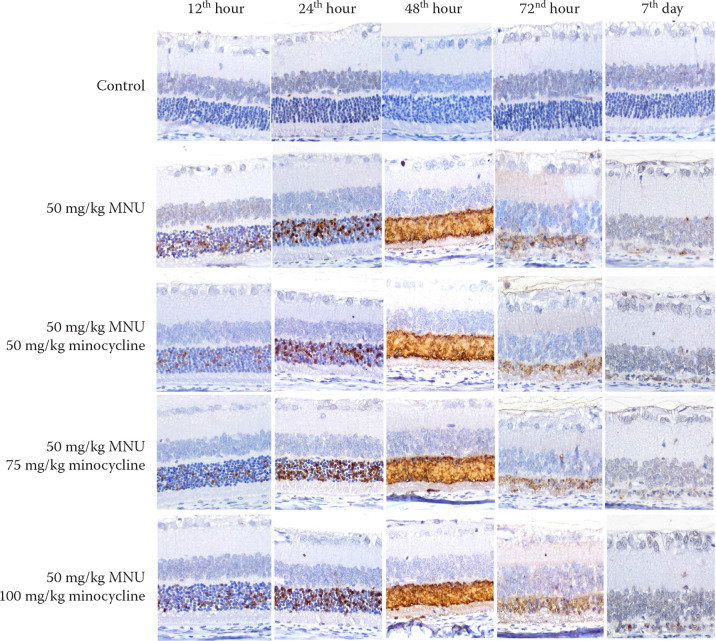
TUNEL positivity in the ONL in all the MNU given groups (brown stained cells) MNU = *N*-methyl-*N*-nitrosourea; ONL = outer nuclear layer; TUNEL = terminal deoxynucleotidyl transferase dUTP nick end labeling

The mean and standard deviation values of the apoptotic indices for all the euthanasia periods are given in Table 4. While the retinas of the control animals were negative in all periods, varying degrees of positivity were seen in the experimental groups (Figure 4). The positivity was only in the photoreceptor cell nuclei located in the ONL of the retina. It was observed that the positivity decreased as it moved from the central to the periphery, and it was completely absent at a distance of approximately 1 500–2 000 μm from the optic nerve. No positivity was observed in the other layers. There was no statistically significant difference between the experimental groups at any of the periods.

**Table 4:** The effects of the minocycline used in three different doses (50, 75 and 100 mg/kg) in the experimental retinal degeneration induced by *N*-methyl-*N*-nitrosourea (MNU) in the rats on the apoptotic indices

Groups	Time					*P*-value
	12^th^ hour	24^th^ hour	48^th^ hour	72^nd^ hour	7^th^ day	
Control	0.00 ± 0.00^B^	0.00 ± 0.00^B^	0.00 ± 0.00^B^	0.00 ± 0.00^B^	0.00 ± 0.00^B^	–
MNU	10.05 **±** 1.66^A,c^	34.61 ± 3.72^A,ab^	93.37 ± 4.29^A,a^	14.92 ± 1.41^A,bc^	12.23 ± 1.17^A,bc^	0.000 1***
LDM	9.80 **±** 1.66^A,d^	35.78 ± 1.76^A,b^	91.07 ± 6.11^A,a^	14.90 ± 1.06^A,c^	10.42 ± 1.84^AB,d^	0.000 1**
MDM	10.01 **±** 1.52^A,d^	33.87 ± 2.09^A,b^	90.39 ± 4.41^A,a^	15.63 ± 1.04^A,c^	11.33 ± 1.48^AB,d^	0.000 1**
HDM	10.03 **±** 1.24^A,d^	35.22 ± 1.15^A,b^	93.61 ± 4.01^A,a^	15.34 ± 0.80^A,c^	10.18 ± 1.80^B,d^	0.000 1**
*P*-value	0.000 1*	0.000 1*	0.000 1*	0.000 1*	0.000 1***	–

Data are given as the mean **±** standard deviation

^a–c^The difference between mean values showing different words in the same line is statistically significant (*P **< ***0.05); ^A–D^The difference between the mean values showing different words in the same column is statistically significant (*P **< ***0.05); *Data were evaluated with a one-way ANOVA and Tukey’s test was used as the post hoc test for the pair comparisons of the groups; **Data were evaluated with Welch’s-ANOVA and Dunnett’s T3 was used as the post hoc test for the pair comparisons of the groups; ***Data were evaluated with the Kruskal-Wallis test and Dunn’s test was used as the post hoc test for the pair comparisons of the groups

HDM = high-dose minocycline; LDM = low-dose minocycline; MDM = medium-dose minocycline

At the 12^th^ hour, the positivity was found to be around 9–10%, with the highest positivity in the MNU group at 10.05%, and the lowest in the LDM group at 9.80%.

At the 24^th^ hour, the number of positive cells and staining intensity increased in the ONL compared to the 12^th^ hour, and the positivity was found to be around 33–35% in the experimental groups. The highest positivity was found in the LDM group at 35.78%, and the lowest positivity was found in the MDM group at 33.87%.

In the experimental group, animals euthanised at the 48^th^ hour showed an increase in the number of positive cells and staining intensity in the ONL compared to the 24^th^ hour. The ONL was selectively stained brown throughout due to the severe and widespread karyorrhexis. The positivity was found to be around 90–93%. The highest positivity was found in the HDM group at 93.61%, and the lowest positivity was found in the MDM group at 90.39%.

At the 72^nd^ hour, positivity was observed in the ONL with 14–15%. The highest positivity was found in the MDM group at 15.63%, and the lowest positivity was found in the LDM group at 14.9%. It was observed that the positivity, which reached the highest level at the 48^th^ hour, decreased significantly due to the decrease in the number of surviving cells.

On the 7^th^ day, the positivity was found at 10–12%. The highest positivity was seen in the MNU group at 12.23%, and the lowest positivity was found in the HDM group at 10.18%.

### Immunohistochemical findings

#### RHODOPSIN

At the 12^th^ and 24^th^ hours, the immunoperoxidase (IP) staining showed widespread and strong positivity in the outer nuclear layer (ONL) and photoreceptor layer (PL) in all the groups. The positivity was intracytoplasmic, and the cell nuclei remained unstained. No difference was noted between the central and peripheral retina, as both exhibited intense staining. There was no statistically significant difference between the groups.

By the 48^th^ hour, the staining intensity in the ONL and PL of the central retina in the experimental groups had decreased, with a particulate and occasionally cavity-like appearance. The staining intensity increased toward the periphery, resembling that of the control group.

**Figure 5:**
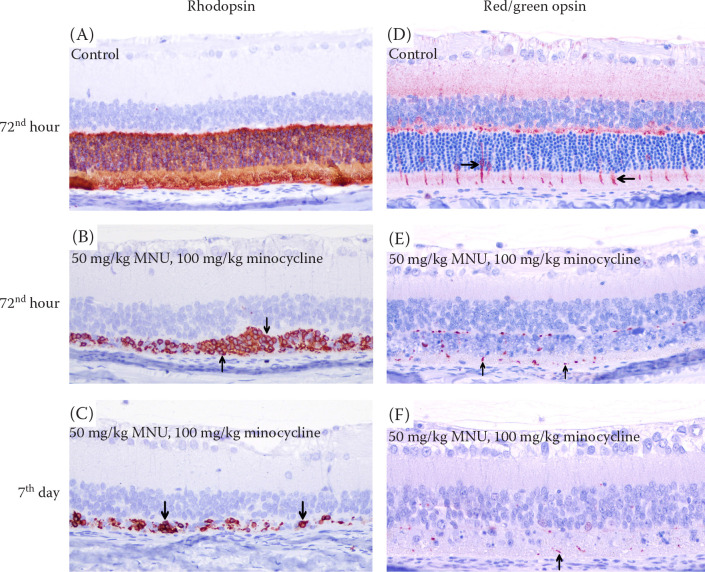
Rhodopsin (A–C) and red/green opsin (D–F) positivity (arrows) in the ONL and PL (72^nd^ hour and 7^th^ day) ONL = outer nuclear layer; PL = photoreceptor layer

At the 72^nd^ hour and on the 7^th^ day, the staining in the central retina of the experimental groups was significantly reduced. Staining was seen only around the surviving photoreceptor cell nuclei, and with no staining in the PL (Figure 5). Statistically significant differences were observed between the control and experimental groups at the 48^th^ and 72^nd^ hours and on the 7^th^ day (*P* < 0.05); no significant differences were found among the experimental groups.

#### RED-GREEN OPSIN

At the 12^th^ and 24^th^ hours, the IP staining showed rare, but strong positivity in the ONL, PL, and the outer plexiform layer (OPL). The positivity was intracytoplasmic, with unstained cell nuclei. The staining began as a long, thin line in the PL, thickened around the photoreceptor nucleus in the ONL, and terminated at the ONL-OPL border (Figure 5). Due to the smaller number of cone receptors compared to rod receptors, positivity appeared at intervals of 15–20 μm. At the 48^th^ and 72^nd^ hours, and on the 7^th^ day, the staining was reduced in all the experimental groups compared to the control group. The previously observed long, thin staining became fragmented and particulate (Figure 5E,F). Statistically significant differences were observed between the control and experimental groups (*P* < 0.05), while no significant differences were detected among the experimental groups.

#### GFAP

**Figure 6:**
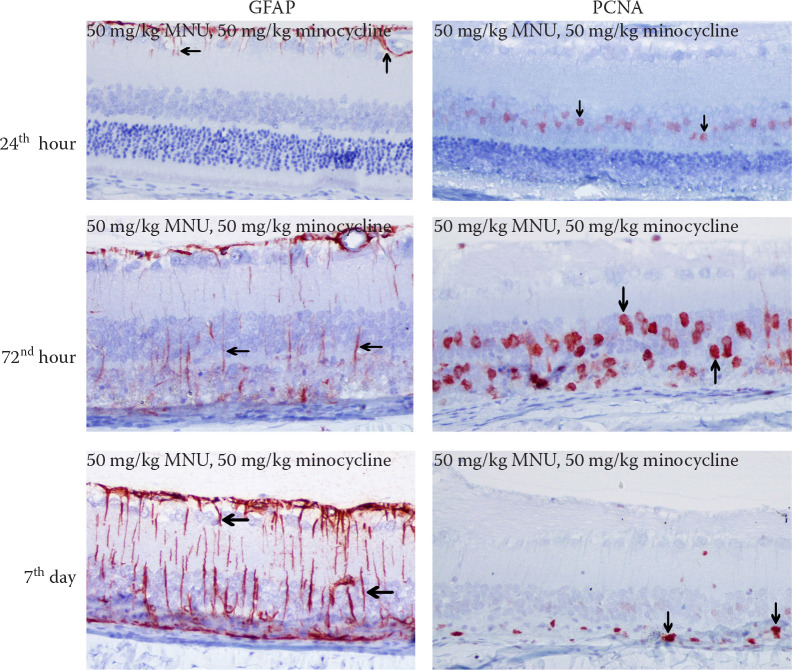
GFAP and PCNA positivity in the retinal layers (arrows) (24^th^, 72^nd^ hours and 7^th^ day) GFAP = glial fibrillary acidic protein; MNU = *N*-methyl-*N*-nitrosourea; PCNA = proliferating cell nuclear antigen

The GFAP staining showed intense positivity in the ganglion cell layer (GCL) and optic nerve across all the groups at the 12^th^ and 24^th^ hours. At the 48^th^ and 72^nd^ hours, the experimental groups also exhibited positivity, like fringes, extending from the GCL to the ONL in a fringe-like pattern, in addition to the optic nerve and GCL. By the 7^th^ day, the positivity had further increased in the experimental groups (Figure 6). Statistically significant differences were observed between the control and experimental groups at the 48^th^ and 72^nd^ hours and on the 7^th^ day, but no differences were found among the experimental groups.

#### PCNA

The PCNA staining showed no positivity in the control group at any of the time points. In the experimental groups, positivity was observed in the inner nuclear layer (INL) at 24 h, and in the INL, ONL, and PL at 48 hours. By the 72^nd^ hour, the positivity further increased, with the Müller cells and macrophages showing staining (Figure 6). By the 7^th^ day, the positivity subsequently decreased. Differences between the control and experimental groups were statistically significant at the 48^th^, 72^nd^ hours, and on the 7^th^ day, but no differences were found among the experimental groups.

### Electron microscopic findings

**Figure 7:**
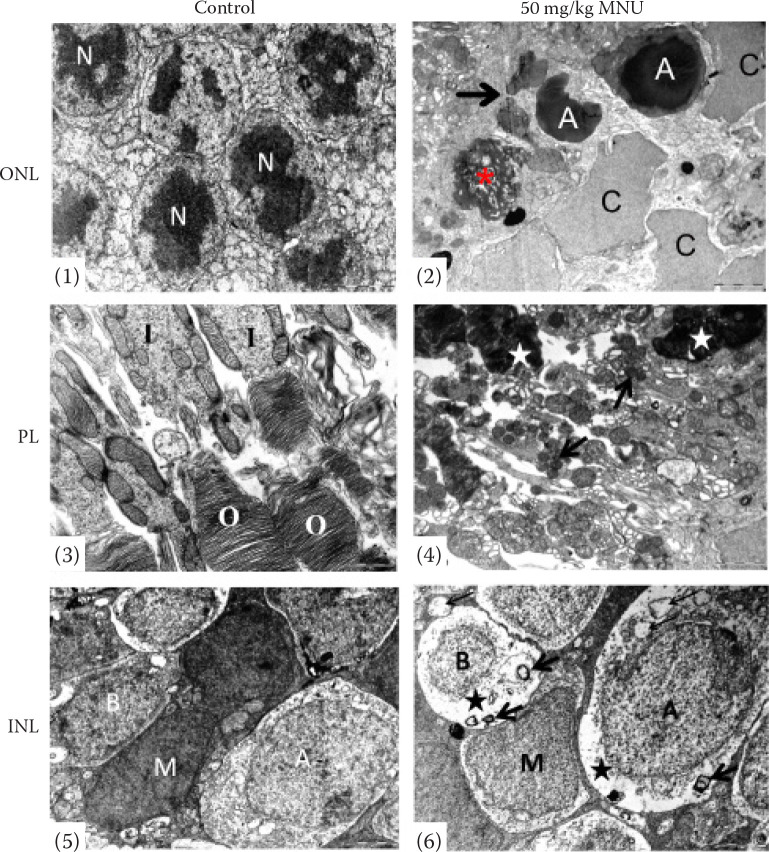
Electron microscopic view of the retina (1) Normal view of photoreceptor nuclei (N). (2) Apoptotic nuclei of the photoreceptors (A), chromatolysis (C), cell degeneration (asterisk), and nuclear fragmentation (arrows) (MNU group, 24^th^ hour). (3) Normal view of the photoreceptor body parts, outer (O) and inner (I) segments. (4) Pycnotic mitochondria (arrows) in the inner segments, condensation in the outer segment discs (stars) of the photoreceptors (MNU group, 24^th^ hour). (5) Normal view bipolar cell (B) Müller cell (M) and amacrine cell (A). (6) Degeneration and intracytoplasmic oedema (stars), myelin figures (thick arrows), dilatation and loss of crystals in mitochondria (thin arrows) in the bipolar cell (B) and amacrine cells (A), normal Müller cell (M) (MNU group, 48^th^ hour) INL = internal nuclear layer; MNU = *N*-methyl-*N*-nitrosourea; ONL = outer nuclear layer; PL = photoreceptor layer

The electron microscopy revealed no significant findings in the control group. At the 12^th^ and 24^th^ hours, the retinal samples from the MNU-treated experimental groups showed nuclear pyknosis as the most prominent finding in the photoreceptor cell nuclei in the ONL. Additionally, nuclear chromatolysis and intracytoplasmic myelin figures were noted. In the PL, pyknotic mitochondria were observed in the inner segments of the rod and cone receptors, with condensation and degenerative changes in the outer segment discs. Bipolar and amacrine cells in the INL were observed to show degenerative changes such as intracytoplasmic oedema, formation of myelin figures, mitochondrial dilatation, and loss of cristae. Müller cells appeared unaffected. At the 48^th^ hour, nuclear condensation and total organelle degeneration were noted in the ONL, along with irregularity and condensation in the outer segment discs (Figure 7). By the 72^nd^ hour and on the 7^th^ day, nuclear condensation, nuclear chromatolysis, and fragmentation were observed in the ONL. Additionally, pyknotic mitochondria, lysozyme deposition, and autophagosomes containing myelin figures were noted in the photoreceptor cells. Degenerative changes, intracytoplasmic oedema, mitochondrial dilatation, and loss of cristae were observed in some bipolar cells.

## DISCUSSION

In similar studies with MNU in the past, chemicals suitable for targeting the pathogenesis of the model have been used as therapeutic agents. These include PARP [Poly (ADP-ribose) polymerase] inhibitors (Kiuchi et al. 2003), calpain inhibitors (Kuro et al. 2011), food supplements (Jing et al. 2023; Shi et al. 2024), neurotropic factors (Xu et al. 2008), heat shock protein activators, such as valproic acid (Koriyama et al. 2014), and antioxidants (Zhou et al. 2022). Positive results were obtained from these studies, especially with antioxidants, as it is known that patients with this condition had high levels of reactive oxygen radicals and low levels of antioxidants, such as glutathione, superoxide dismutase, and catalase, compared to healthy subjects (Danulescu and Costin 2012). However, the results remain limited in their effectiveness in preventing RD, and it is still important to find new substances or combinations that are likely to be effective.

Tsubara et al. (2007), in their studies using a single dose of 50–75 mg/kg of MNU, showed that histopathologically, at the 24^th^ hour, the photoreceptor nuclei were hyperchromatic stained; at the 48^th^ hour, the photoreceptor cells showed intense pyknosis and karyorrhexis; at the 7^th^ day, the ONL and PL almost disappeared and the INL came into contact with the choroid and destruction in areas, close to the optic nerve, was more severe. In the present study, similar findings were obtained histopathologically, but two different conditions were identified. The first is the absence of hyperchromatic staining of the photoreceptor nuclei at 24 h, since it is thought to be difficult to detect hyperchromasia in these cells, which are already strongly hyperchromatic. The second difference is that the peripheral retina of a small number of animals was also significantly affected. Here, it is thought that individual differences between animals may influence resistance to MNU.

Tsubara et al. (2007) demonstrated that the most typical finding from the 12^th^ hour was the condensed photoreceptor nuclei in electron microscopic examinations of MNU-induced RD. In the present study, condensation of photoreceptor nuclei was the most striking finding. As an important point here, the degeneration of amacrine and bipolar cells, only seen in electron microscopy, differed from the claim that MNU affects only photoreceptors.

As a therapeutic agent, minocycline has not been previously used in RD models induced by MNU, but has been used in light-induced RD (Zhang et al. 2004; Chang et al. 2005), transgenic animals with a genetic defect causing RD (Peng et al. 2014; Terauchi et al. 2021; Ozaki et al. 2022), in patients with age-related degeneration (Sivaprasad et al. 2011; Du et al. 2022), in mice with retinal detachment (Yang et al. 2009), glaucoma models (Grotegut et al. 2020) and mouse models with subretinal bleeding (Zhao et al. 2011), with varying degrees of positive results. In these studies, the anti-apoptotic and anti-inflammatory activities (Thomas et al. 2003), which are completely independent of the antimicrobial activity of minocycline, and the ability to cross the blood-brain barrier (Bonelli et al. 2003), came to the fore. In particular, neuroinflammation is believed to play a role in the pathogenesis of many chronic neurodegenerative diseases (Glass et al. 2010). Past studies have shown that microglial activation is associated with hereditary retinal degeneration (Langman 2007).

In RD studies, minocycline was administered daily either before, after, or during the experiment (Zhao et al. 2011; Peng et al. 2014). In this study, minocycline was administered in two doses, 24 h before and immediately after the MNU administration. Daily dosing was avoided because euthanasia would begin after the 12^th^ hour. To determine if the efficacy of minocycline was dose-dependent, it was administered at 50, 75, or 100 mg/kg. Despite some success in RD models with light exposure and transgenic animals, minocycline did not achieve the expected results in the MNU-induced model. The ineffectiveness of minocycline in the MNU-induced RD model is thought to result from factors such as the involvement of multiple caspases, including caspase-3, -6, -8, -9, and -12 (Yoshizawa et al. 1999), DNA alkylation by MNU occurring too early, between 6 and 12 h (Ogino et al. 1993), and the possible predominance of caspase-independent (calpain-dependent) apoptosis (Reisenhofer et al. 2015).

In conclusion, based on the data from this study, it can be stated that a single dose of MNU induced severe photoreceptor apoptosis quickly in the RD model, which is beneficial for gathering information on RD-related diseases. While minocycline is known to be effective against neurodegenerative diseases, it was found to be ineffective in this particular model. Although many studies have demonstrated the anti-apoptotic properties of minocycline, the complex nature of MNU-induced retinal degeneration may have limited its effectiveness. Future research could explore the use of a longer-term MNU model with daily administration of 1–2 doses of minocycline or investigate the potential of combining minocycline with other drugs.
